# 3-(2-Chloro­ethyl)-2-methyl-4*H*-pyrido[1,2-*a*]pyrimidin-4-one

**DOI:** 10.1107/S1600536809027548

**Published:** 2009-07-25

**Authors:** Jerry P. Jasinski, Ray J. Butcher, Q. N. M. Hakim Al-Arique, H. S. Yathirajan, B. Narayana

**Affiliations:** a Department of Chemistry, Keene State College, 229 Main Street, Keene, NH 03435-2001, USA; b Department of Chemistry, Howard University, 525 College Street NW, Washington DC 20059, USA; c Department of Studies in Chemistry, University of Mysore, Manasagangotri, Mysore 570 006, India; d Department of Studies in Chemistry, Mangalore University, Mangalagangotri 574 199, India

## Abstract

In the title mol­ecule, C_11_H_11_ClN_2_O, the pyrido[1,2-*a*]pyrimidine ring system is planar (maximum deviation = 0.0148 Å) and the methyl C and carbonyl O atoms are nearly coplanar to it. The chloro­ethyl side chain is in a synclinal conformation, nearly orthogonal to the pyrimidine ring, with a dihedral angle between the chloro­ethyl side chain and the pyrimidine ring of 88.5 (1)°. Weak inter­molecular C—H⋯N and C—H⋯Cl hydrogen bonds along with π–π inter­actions between the pyrimidine and pyridine rings [centroid–centroid distance is 3.538 (2) Å] form a three-dimensional network. The crystal is a racemic twin with a 0.68 (12):0.32 (12) domain ratio. MOPAC AM1 and density functional theory (DFT) theoretical calculations at the B3-LYP/6–311+G(d,p) level support these observations.

## Related literature

For related structures, see: Blaton *et al.* (1995 ▶); Chen & He (2006 ▶); Elotmani *et al.* (2002 ▶); Jottier *et al.* (1992 ▶); Koval’chukova *et al.* (2004 ▶); Peeters *et al.* (1993 ▶); Ravikumar & Sridhar, (2006 ▶); Yu *et al.* (2007 ▶). For general background to heterofused pyrimidines, see: Baraldi *et al.* (2002 ▶); Bookser *et al.* (2005 ▶); Chen *et al.* (2004 ▶); La Motta *et al.* (2007 ▶); Gabbert & Giannini (1997 ▶); Goodacre *et al.* (2006 ▶); Hossain *et al.* (1997 ▶); Joseph & Burke (1993 ▶); Nikitin & Smirnov (1994 ▶); Sabnis & Rangnekar (1990 ▶); Wang *et al.* (2004 ▶); White *et al.* (2004 ▶). For the synthesis, see: Toche *et al.* (2008 ▶). For *GAUSSIAN03* theoretical calculations, see: Becke (1988 ▶, 1993 ▶); Frisch *et al.* (2004 ▶); Hehre *et al.* (1986 ▶); Lee *et al.* (1988 ▶); Schmidt & Polik (2007 ▶).
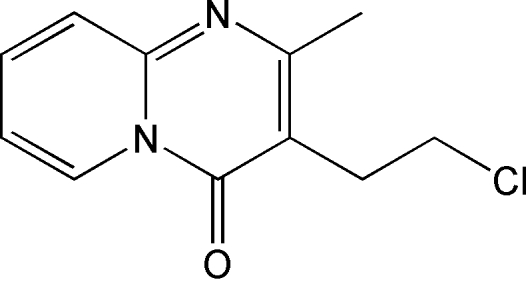


## Experimental

### 

#### Crystal data

C_11_H_11_ClN_2_O
*M* *_r_* = 222.67
Orthorhombic, 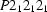

*a* = 4.2546 (4) Å
*b* = 11.6274 (10) Å
*c* = 20.604 (2) Å
*V* = 1019.27 (17) Å^3^
*Z* = 4
Mo *K*α radiation
μ = 0.35 mm^−1^
*T* = 110 K
0.51 × 0.35 × 0.12 mm

#### Data collection

Oxford Diffraction Gemini R CCD diffractometer
Absorption correction: multi-scan (*CrysAlis RED*; Oxford Diffraction, 2007 ▶) *T*_min_ = 0.835, *T*_max_ = 0.959
4613 measured reflections
3089 independent reflections
2607 reflections with *I* > 2σ(*I*)
*R*_int_ = 0.054

#### Refinement

*R*[*F* ^2^ > 2σ(*F* ^2^)] = 0.065
*wR*(*F* ^2^) = 0.181
*S* = 1.11
3089 reflections
138 parameters
H-atom parameters constrained
Δρ_max_ = 0.99 e Å^−3^
Δρ_min_ = −0.52 e Å^−3^
Absolute structure: Flack (1983 ▶), 1103 Friedel pairs
Flack parameter: 0.32 (12)

### 

Data collection: *CrysAlisPro* (Oxford Diffraction, 2007 ▶); cell refinement: *CrysAlisPro*; data reduction: *CrysAlisPro*; program(s) used to solve structure: *SHELXS97* (Sheldrick, 2008 ▶); program(s) used to refine structure: *SHELXL97* (Sheldrick, 2008 ▶); molecular graphics: *SHELXTL* (Sheldrick, 2008 ▶); software used to prepare material for publication: *SHELXTL*.

## Supplementary Material

Crystal structure: contains datablocks global, I. DOI: 10.1107/S1600536809027548/ci2827sup1.cif

Structure factors: contains datablocks I. DOI: 10.1107/S1600536809027548/ci2827Isup2.hkl

Additional supplementary materials:  crystallographic information; 3D view; checkCIF report

## Figures and Tables

**Table 1:** Hydrogen-bond geometry (Å, °)

*D*—H⋯*A*	*D*—H	H⋯*A*	*D*⋯*A*	*D*—H⋯*A*
C5—H5*A*⋯N2^i^	0.95	2.50	3.394 (3)	157
C2—H2*A*⋯Cl^ii^	0.95	2.90	3.559 (3)	128

Symmetry codes: (i) 
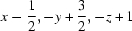
; (ii) 
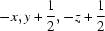
.

**Fig. 1.**
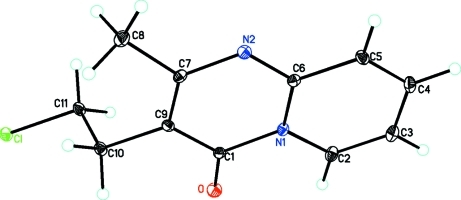
Molecular structure of (I), showing the atom labeling scheme and 50% probability displacement ellipsoids.

**Fig. 2.**
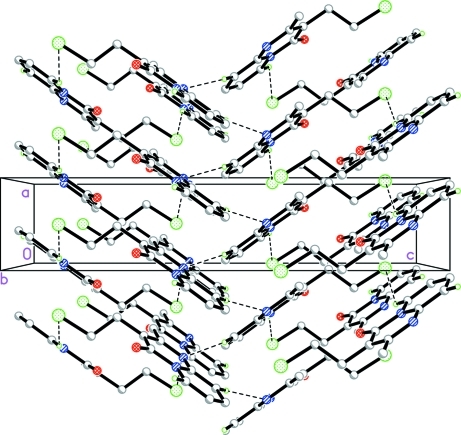
Packing diagram of (I), viewed down the b axis. Dashed lines indicate weak C5—H5A···N2 and C2—H2A···Cl intermolecular interactions.

## supplementary crystallographic information

### Comment

Heterofused pyrimidines exhibit promising antiviral (Hossain *et al.* 1997), antibacterial (Sabnis & Rangnekar, 1990), anti-AIDS (Joseph & Burke, 1993), and antinociceptive (Bookser *et al.* 2005) activities. Fused pyrimidines are extensively used in neurology, particularly in the treatment of neurodegenerative disorders such as Parkinson's disease (Baraldi *et al.* 2002), antianxiety disorders (Goodacre *et al.* 2006) and depression (Chen *et al.* 2004). Fused pyrimidines are selective inhibitors for multidrug resistance (MDR) (Wang *et al.* 2004). A review on the synthesis, chemical and biological properties of pyrido[1,2-*a*]pyrimidines is described (Nikitin & Smirnov, 1994). Pyrido[1,2-*a*]pyrimidin-4-one derivatives as a novel class of selective aldose reductase inhibitors exhibiting antioxidant activity has been reported (La Motta *et al.* 2007). The synthesis and anticonvulsant evaluation of some new 2-substituted-3-arylpyrido[2,3-*d*]pyrimidinones have also been reported (White *et al.* 2004). The crystal structures of 3-{2-[4-(6-fluoro-1,2-benzisoxazol-3-yl)piperidino]ethyl}-6,7,8,9- tetrahydro-2-methyl-4*H*-pyrido[1,2-*a*]pyrimidin-4-one (risperidone) (Peeters *et al.* 1993), 3-{2-[4-(4-fluorobenzoyl)piperidino]ethyl}-2-methyl-4*H*-pyrido[1,2-*a*]pyrimidin-4-one (Pirenperone) (Blaton *et al.* 1995), 5-methyl-2-morpholino-3-*p*-tolyl-8,9,10,11-tetrahydro-2-benzothieno[2', 3':6,5]pyrido[4,3-*d*]pyrimidin-4(3*H*)-one (Chen & He, 2006), 3-{2-[4-(6-fluoro-1,2-benzisoxazol-3-yl)-1-piperidinyl]ethyl}-2,9- dimethyl-4*H*-pyrido[1,2-*a*]pyrimidin-4-one (Ocaperidone) (Jottier *et al.* 1992), 2-methyl-3-(3-methyl-1*H*-pyrazol-5-yl)pyrido[1,2-*a*]pyrimidin-4-one (Elotmani *et al.* 2002), 2-methyl-3-chloro-9-hydroxypyrido[1,2-*a*]pyrimidin-4-one and bis(2-methyl-3-chloro-9-hydroxypyrido[1,2-*a*]pyrimidin-4-onium) perchlorate (Koval'chukova *et al.* 2004), 3-(2-chloroethyl)-2-methyl-4-oxo-6,7,8,9-tetrahydro-4*H*-pyrido[1,2-*a*] pyrimidin-1-ium chloride (Ravikumar & Sridhar, 2006) and 9-(4-methoxybenzoyl)-1,2,3,4-tetrahydro-6*H*-pyrido[1,2-*a*]pyrimidin-6-one (Yu *et al.* 2007) have also been reported.

The title compound, (I), is an intermediate in the synthesis of risperidone, which is a potent antipsychotic agent, especially useful for treating schizophrenia (Gabbert & Giannini, 1997). In view of the importance of (I), the present paper describes its crystal structure.

The overall molecular geometry of (I), including bond distances and angles, is in good agreement with related structures (Blaton *et al.* 1995; Jottier *et al.* 1992; Peeters *et al.* 1993; Ravikumar & Sridhar, 2006). It consists of a pyridine ring fused to a substituted pyrimidine ring creating a planar ring system (maximum deviation, C1, = -0.0148Å) with the methyl C and carbonyl O atoms nearly coplanar to the pyrimidine ring (Torsion angles C1-C9-C7-C8 = 177.6 (3)° ; C2-N1-C1-O = -2.5 (4)° (Fig. 1). The sum of the angles aroumd N1 is 360.0 (5)° indicating *sp*^2^ hybridization. The chloroethyl side chain is in a synclinal (-*sc*) conformation (C1—C9—C10—C11 torsion angle = -86.6 (3)°), nearly orthogonal to the pyrimidine ring, with a dihedral angle separation between the C10/C11/Cl group and the pyrimidine ring of 88.5 (1)°.

While no classic hydrogen bonds are observed, a weak intermolecular hydrogen bond interaction exists between atom C5 from the pyridine ring and N2 from a nearby pyrimidine ring (Table 1 and Fig. 2). In addition, a weak intermolecular interaction between atom C2 from the pyrimidine ring and Cl from the substituted pyrimidine group also occurs, each influencing crystal packing and, therefore, resulting in a three-dimensional network (Fig. 2). In addition, π-π interactions between N1/C1/C9/C7/N2/C6 (centroid Cg1) and N1/C2-C6 (centroid Cg2) rings of molecules at (x, y, z) and (1+x, y, z), with a Cg1···Cg2 distance of 3.538 (2) Å, provide additional stability to the crystal packing. The crystal is a racemic twin with domains of 0.68 (12) and 0.32 (12).

In support of these observations, a MOPAC AM1 (Schmidt & Polik, 2007) and density functional theory (DFT) geometry optimized theoretical calculation (Schmidt & Polik, 2007) with the *GAUSSIAN03* program package (Frisch *et al.* 2004) employing the B3-LYP (Becke 3 parameter Lee-Yang-Parr) exchange correlation functional, which combines the hybrid exchange functional of Becke (Becke, 1988, 1993) with the gradient-correlation functional of Lee, Yang and Parr (Lee *et al.* 1988) and the 6–311+G(d,p) basis set (Hehre *et al.* 1986), was performed on (I) utilizing starting geometries taken from the X-ray refinement data. In both calculations the resulting bond distances and angles remained relatively constant. However, the C9—C10—C11—Cl torsion angle decreased by 3.2 (1)° to 175.4 (3)° (MOPAC) and 0.07° to 178.5 (7)° (DFT) and the dihedral angle between the C10/C11/Cl group and the pyrimidine ring decreased by 2.3 (8)° to 86.1 (3)° (MOPAC) and by 8.3 (6)° to 80.1 (5)° (DFT), respectively.

In summary, it is clear that the collection of weak intermolecular hydrogen bond interactions and π-π intermolecular interactions do play a role in stabilizing crystal packing of (I).

### Experimental

The title compound was synthesized following the reported procedure (Toche *et al.* 2008). Pale yellow crystals of compound (I) were obtained by slow evaporation from ethyl acetate solution (m.p. 405–408 K). Analytical data: Found (calculated): C %: 59.28 (59.33); H%: 4.97 (4.98); N%: 12.54 (12.58).

### Refinement

All of the H atoms were placed in their calculated positions and then refined using the riding model with C—H = 0.95–0.99 Å, and with *U*_iso_(H) = 1.18–1.50*U*_eq_(C).

### Crystal data

**Table:**

C_11_H_11_ClN_2_O	*F*(000) = 464
*M**_r_* = 222.67	*D*_x_ = 1.451 Mg m^−^^3^
Orthorhombic, *P*2_1_2_1_2_1_	Mo *K*α radiation, λ = 0.71073 Å
Hall symbol: P 2ac 2ab	Cell parameters from 2755 reflections
*a* = 4.2546 (4) Å	θ = 4.8–32.6°
*b* = 11.6274 (10) Å	µ = 0.35 mm^−^^1^
*c* = 20.604 (2) Å	*T* = 110 K
*V* = 1019.27 (17) Å^3^	Plate, colorless
*Z* = 4	0.51 × 0.35 × 0.12 mm

### Data collection

**Table:**

Oxford Diffraction Gemini R CCD diffractometer	3089 independent reflections
Radiation source: Enhance (Mo) X-ray Source	2607 reflections with *I* > 2σ(*I*)
graphite	*R*_int_ = 0.054
Detector resolution: 10.5081 pixels mm^-1^	θ_max_ = 32.6°, θ_min_ = 4.9°
φ and ω scans	*h* = −3→6
Absorption correction: multi-scan (*CrysAlis RED*; Oxford Diffraction, 2007)	*k* = −17→16
*T*_min_ = 0.835, *T*_max_ = 0.959	*l* = −30→27
4613 measured reflections	

### Refinement

**Table:**

Refinement on *F*^2^	Secondary atom site location: difference Fourier map
Least-squares matrix: full	Hydrogen site location: inferred from neighbouring sites
*R*[*F*^2^ > 2σ(*F*^2^)] = 0.065	H-atom parameters constrained
*wR*(*F*^2^) = 0.181	*w* = 1/[σ^2^(*F*_o_^2^) + (0.1108*P*)^2^ + 0.2652*P*] where *P* = (*F*_o_^2^ + 2*F*_c_^2^)/3
*S* = 1.11	(Δ/σ)_max_ = 0.001
3089 reflections	Δρ_max_ = 0.99 e Å^−^^3^
138 parameters	Δρ_min_ = −0.51 e Å^−^^3^
0 restraints	Absolute structure: Flack (1983), 1103 Friedel pairs
Primary atom site location: structure-invariant direct methods	Flack parameter: 0.32 (12)

### Special details

**Table:**

Geometry. All e.s.d.'s (except the e.s.d. in the dihedral angle between two l.s. planes) are estimated using the full covariance matrix. The cell e.s.d.'s are taken into account individually in the estimation of e.s.d.'s in distances, angles and torsion angles; correlations between e.s.d.'s in cell parameters are only used when they are defined by crystal symmetry. An approximate (isotropic) treatment of cell e.s.d.'s is used for estimating e.s.d.'s involving l.s. planes.
Refinement. Refinement of *F*^2^ against ALL reflections. The weighted *R*-factor *wR* and goodness of fit *S* are based on *F*^2^, conventional *R*-factors *R* are based on *F*, with *F* set to zero for negative *F*^2^. The threshold expression of *F*^2^ > σ(*F*^2^) is used only for calculating *R*-factors(gt) *etc*. and is not relevant to the choice of reflections for refinement. *R*-factors based on *F*^2^ are statistically about twice as large as those based on *F*, and *R*- factors based on ALL data will be even larger.

### Fractional atomic coordinates and isotropic or equivalent isotropic displacement parameters (Å^2^)

**Table:**

	*x*	*y*	*z*	*U*_iso_*/*U*_eq_	
Cl	0.49448 (19)	0.87358 (6)	0.12093 (3)	0.02056 (18)	
O	0.1937 (6)	1.09071 (18)	0.30555 (10)	0.0240 (5)	
N1	−0.0403 (6)	0.9961 (2)	0.39118 (10)	0.0150 (4)	
N2	0.0097 (7)	0.79306 (19)	0.40322 (11)	0.0172 (4)	
C1	0.1541 (7)	0.9982 (2)	0.33357 (13)	0.0151 (5)	
C2	−0.1616 (9)	1.0994 (2)	0.41318 (14)	0.0207 (6)	
H2A	−0.1116	1.1685	0.3909	0.025*	
C3	−0.3506 (8)	1.1032 (3)	0.46596 (15)	0.0231 (6)	
H3A	−0.4354	1.1744	0.4803	0.028*	
C4	−0.4212 (7)	0.9999 (3)	0.49970 (14)	0.0211 (6)	
H4A	−0.5530	1.0019	0.5369	0.025*	
C5	−0.3006 (8)	0.8986 (2)	0.47886 (13)	0.0191 (5)	
H5A	−0.3477	0.8299	0.5019	0.023*	
C6	−0.1033 (7)	0.8935 (2)	0.42262 (13)	0.0147 (5)	
C7	0.1973 (8)	0.7917 (2)	0.34977 (13)	0.0155 (5)	
C8	0.3087 (9)	0.6735 (2)	0.33069 (16)	0.0239 (6)	
H8A	0.2581	0.6188	0.3653	0.036*	
H8B	0.5366	0.6750	0.3238	0.036*	
H8C	0.2038	0.6499	0.2905	0.036*	
C9	0.2748 (7)	0.8891 (2)	0.31499 (12)	0.0146 (5)	
C10	0.4766 (9)	0.8869 (2)	0.25437 (12)	0.0182 (5)	
H10A	0.6268	0.8220	0.2567	0.022*	
H10B	0.5983	0.9592	0.2511	0.022*	
C11	0.2671 (7)	0.8733 (3)	0.19491 (13)	0.0201 (5)	
H11A	0.1130	0.9371	0.1936	0.024*	
H11B	0.1489	0.8002	0.1982	0.024*	

### Atomic displacement parameters (Å^2^)

**Table:**

	*U*^11^	*U*^22^	*U*^33^	*U*^12^	*U*^13^	*U*^23^
Cl	0.0233 (3)	0.0253 (3)	0.0132 (3)	−0.0031 (3)	0.0030 (3)	−0.0001 (2)
O	0.0323 (13)	0.0185 (9)	0.0214 (10)	−0.0029 (9)	0.0024 (10)	0.0048 (8)
N1	0.0188 (11)	0.0144 (8)	0.0118 (9)	0.0013 (9)	−0.0009 (9)	0.0005 (7)
N2	0.0200 (10)	0.0157 (8)	0.0160 (10)	−0.0002 (12)	−0.0001 (11)	0.0019 (7)
C1	0.0168 (12)	0.0163 (11)	0.0122 (11)	−0.0028 (11)	−0.0005 (10)	0.0002 (9)
C2	0.0286 (15)	0.0163 (11)	0.0173 (13)	0.0039 (12)	0.0000 (12)	−0.0001 (9)
C3	0.0259 (15)	0.0235 (13)	0.0198 (14)	0.0058 (13)	−0.0013 (12)	−0.0054 (10)
C4	0.0198 (14)	0.0300 (14)	0.0135 (12)	0.0026 (12)	0.0017 (10)	−0.0019 (10)
C5	0.0196 (13)	0.0234 (12)	0.0142 (12)	−0.0029 (12)	0.0018 (11)	0.0028 (10)
C6	0.0178 (11)	0.0146 (11)	0.0118 (11)	−0.0013 (9)	−0.0029 (9)	0.0017 (8)
C7	0.0194 (13)	0.0125 (10)	0.0146 (11)	0.0001 (11)	−0.0022 (11)	−0.0001 (9)
C8	0.0282 (16)	0.0167 (11)	0.0267 (15)	0.0015 (13)	0.0028 (14)	−0.0022 (10)
C9	0.0133 (11)	0.0183 (11)	0.0124 (12)	−0.0020 (10)	−0.0004 (9)	0.0005 (9)
C10	0.0164 (12)	0.0245 (12)	0.0138 (11)	−0.0008 (13)	0.0011 (10)	0.0003 (9)
C11	0.0178 (12)	0.0305 (13)	0.0119 (11)	−0.0035 (12)	0.0019 (10)	−0.0012 (11)

### Geometric parameters (Å, °)

**Table:**

Cl—C11	1.805 (3)	C5—C6	1.432 (4)
O—C1	1.232 (3)	C5—H5A	0.95
N1—C2	1.383 (4)	C7—C9	1.381 (4)
N1—C6	1.384 (3)	C7—C8	1.505 (4)
N1—C1	1.447 (3)	C8—H8A	0.98
N2—C6	1.325 (3)	C8—H8B	0.98
N2—C7	1.360 (4)	C8—H8C	0.98
C1—C9	1.421 (4)	C9—C10	1.516 (4)
C2—C3	1.353 (5)	C10—C11	1.523 (4)
C2—H2A	0.95	C10—H10A	0.99
C3—C4	1.420 (5)	C10—H10B	0.99
C3—H3A	0.95	C11—H11A	0.99
C4—C5	1.355 (4)	C11—H11B	0.99
C4—H4A	0.95		
			
C2—N1—C6	121.5 (2)	N2—C7—C8	114.0 (2)
C2—N1—C1	117.9 (2)	C9—C7—C8	122.6 (3)
C6—N1—C1	120.6 (2)	C7—C8—H8A	109.5
C6—N2—C7	117.9 (2)	C7—C8—H8B	109.5
O—C1—C9	127.1 (3)	H8A—C8—H8B	109.5
O—C1—N1	118.5 (3)	C7—C8—H8C	109.5
C9—C1—N1	114.4 (2)	H8A—C8—H8C	109.5
C3—C2—N1	120.9 (3)	H8B—C8—H8C	109.5
C3—C2—H2A	119.5	C7—C9—C1	120.4 (2)
N1—C2—H2A	119.5	C7—C9—C10	123.3 (2)
C2—C3—C4	119.4 (3)	C1—C9—C10	116.3 (2)
C2—C3—H3A	120.3	C9—C10—C11	109.5 (3)
C4—C3—H3A	120.3	C9—C10—H10A	109.8
C5—C4—C3	120.0 (3)	C11—C10—H10A	109.8
C5—C4—H4A	120.0	C9—C10—H10B	109.8
C3—C4—H4A	120.0	C11—C10—H10B	109.8
C4—C5—C6	120.9 (3)	H10A—C10—H10B	108.2
C4—C5—H5A	119.5	C10—C11—Cl	111.4 (2)
C6—C5—H5A	119.5	C10—C11—H11A	109.3
N2—C6—N1	123.3 (2)	Cl—C11—H11A	109.3
N2—C6—C5	119.6 (2)	C10—C11—H11B	109.3
N1—C6—C5	117.2 (2)	Cl—C11—H11B	109.3
N2—C7—C9	123.4 (2)	H11A—C11—H11B	108.0
			
C2—N1—C1—O	−2.5 (4)	C4—C5—C6—N2	−179.6 (3)
C6—N1—C1—O	177.1 (3)	C4—C5—C6—N1	0.5 (4)
C2—N1—C1—C9	178.7 (3)	C6—N2—C7—C9	−0.1 (4)
C6—N1—C1—C9	−1.8 (4)	C6—N2—C7—C8	−178.8 (3)
C6—N1—C2—C3	−0.9 (5)	N2—C7—C9—C1	−1.0 (4)
C1—N1—C2—C3	178.6 (3)	C8—C7—C9—C1	177.6 (3)
N1—C2—C3—C4	1.0 (5)	N2—C7—C9—C10	−178.2 (3)
C2—C3—C4—C5	−0.3 (5)	C8—C7—C9—C10	0.4 (5)
C3—C4—C5—C6	−0.5 (5)	O—C1—C9—C7	−176.9 (3)
C7—N2—C6—N1	0.2 (4)	N1—C1—C9—C7	1.8 (4)
C7—N2—C6—C5	−179.7 (3)	O—C1—C9—C10	0.6 (4)
C2—N1—C6—N2	−179.7 (3)	N1—C1—C9—C10	179.3 (2)
C1—N1—C6—N2	0.8 (4)	C7—C9—C10—C11	90.7 (3)
C2—N1—C6—C5	0.2 (4)	C1—C9—C10—C11	−86.6 (3)
C1—N1—C6—C5	−179.3 (3)	C9—C10—C11—Cl	178.6 (2)

### Hydrogen-bond geometry (Å, °)

**Table:**

*D*—H···*A*	*D*—H	H···*A*	*D*···*A*	*D*—H···*A*
C5—H5A···N2^i^	0.95	2.50	3.394 (3)	157
C2—H2A···Cl^ii^	0.95	2.90	3.559 (3)	128

Symmetry codes: (i) *x*−1/2, −*y*+3/2, −*z*+1; (ii) −*x*, *y*+1/2, −*z*+1/2.
